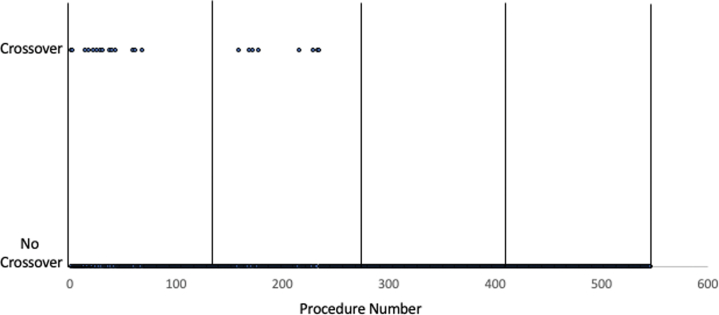# Learning Curve of Robotic Percutaneous Coronary Intervention: A Single-Center Experience

**DOI:** 10.1016/j.jscai.2022.100508

**Published:** 2022-10-13

**Authors:** Tejas M. Patel, Sanjay C. Shah, Aman T. Patel, Bhavin Patel, Samir B. Pancholy

**Affiliations:** a Apex Heart Institute, Ahmedabad, India; b Veterans Administration Medical Center, Wilkes-Barre, Pennsylvania

**Keywords:** learning curve, percutaneous coronary intervention, robotics

## Abstract

**Background:**

Robotic percutaneous coronary intervention (R-PCI) has been shown to provide benefits to operators and patients when compared with traditional percutaneous coronary intervention. Despite being available for 16 years in the United States, utilization of R-PCI remains low. This may be because of an expected learning curve with this technology. We sought to describe the characteristics and magnitude of the learning curve with R-PCI.

**Methods:**

Consecutive patients undergoing R-PCI (Corpath GRX-2) at a tertiary care center by a single operator were studied prospectively. Demographic, angiographic, and procedural variables were collected. The primary study endpoints included fluoroscopy time, procedure time, and contrast volume. The distributions of each of these variables were plotted against the case numbers in chronological sequence, and the best curve fits were identified. Using the best model, the slope of the relationships was analyzed. Flattening of the slope of these plots were considered suggestive of a learning effect.

**Results:**

A total of 546 R-PCI and 1654 traditional percutaneous coronary intervention procedures were studied; 22 crossovers to traditional percutaneous coronary intervention occurred. Most of the crossovers occurred in the first quartile of procedures; no crossovers occurred in the latter half of the cohort. Procedure time decreased as the procedure number increased, with the slope flattening at procedure number 50. Contrast volume decreased as experience increased, with a slope flattening at procedure number 30. Both parameters continued to decrease as experience increased. Fluoroscopy time demonstrated a flattening slope after procedure number 15. This likely is driven by the lower complexity by Synergy between Percutaneous Coronary Intervention with Taxus and Cardiac Surgery (SYNTAX) score remained stable over the procedure sequence, with no significant complexity change over the study period.

**Conclusion:**

The “learning effect” of R-PCI is observed with steep improvement in study metrics up to 50 procedures and a continuing improvement of lesser magnitude afterward.

## Highlights

• Robotic percutaneous coronary intervention has been applied to a variety of interventional subsets, with established benefits for the operator and possibly the patient.
• The learning curve of robotic coronary intervention appears to be around 50 procedures.
• A larger, multicenter, multioperator dataset would be needed to corroborate these findings.

## Central Illustration

**Figure:**
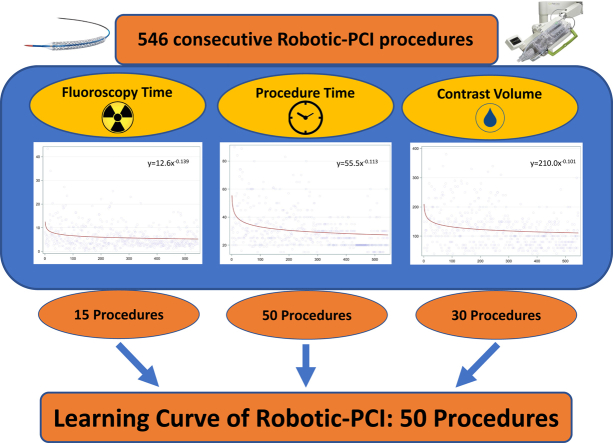

## Introduction

Robotic percutaneous coronary intervention (R-PCI) has been available in the interventional cardiac catheterization laboratory since 2006 in the United States and since 2017 outside the United States. R-PCI enables the operator to control interventional hardware in the patient’s vascular system using a robotic arm mounted on the cardiac catheterization laboratory table while sitting away from the angiographic console. As expected, this reduces operator radiation exposure and eliminates the need for operators to wear heavy shielding garments, potentially reducing an orthopedic hazard.^1^^,^^2^ In a propensity-matched analysis, a signal of reduction in overall radiation utilization was observed with a potential reduction in patient radiation exposure.^3^ Despite these benefits, the utilization of R-PCI remains low. Potential barriers to wider adoption of robotic technology in the cardiac catheterization laboratory may include cost, inability to perform diagnostic coronary angiography as well as ability to use adjunctive devices such as intracoronary imaging catheters, atherectomy, and multiple catheters simultaneously.

In view of a significant change in the ergonomics of the procedure from an operator and team standpoint, a learning process is expected. No assessment of the learning curve of R-PCI is currently available in the literature. Evaluating the threshold to overcome the learning curve could be important for increasing the adoption of R-PCI, improving patient outcomes, and drafting training guidelines for R-PCI. We present a single-center analysis of the learning curve of R-PCI, addressing the commonly used metrics affected by the learning process.

## Patients and methods

Data on patients undergoing R-PCI at Apex Heart Institute, Ahmedabad, India, performed by an experienced interventional operator using Corpath GRX-2 (Corindus) were collected in a prospective registry. All patients receiving R-PCI at Apex Heart Institute were included in the registry. The registry process was approved by the local institutional review board. The primary operator was a highly experienced interventional cardiologist with >30 years of experience in traditional percutaneous coronary intervention (T-PCI), and the secondary operators were physicians with >10 years of experience. Demographic variables including patients’ age, sex, presence of comorbidities including diabetes mellitus, hypertension, previous coronary bypass surgery, and acute coronary syndrome presentation were collected on all patients (Table 1). Procedural variables including total procedure time, contrast volume, fluoroscopy time (FT), air kerma at the interventional reference point, dose-area product, number of catheters used, number of lesions treated, and Synergy between Percutaneous Coronary Intervention with Taxus and Cardiac Surgery (SYNTAX) score were recorded.^4^ Similar details of nonrobotic, T-PCI procedures performed during the study period by multiple operators at the same center were recorded. The primary outcome measures included procedure time, contrast volume, and FT. Secondary outcomes included procedural crossover from R-PCI to T-PCI, defined as the necessity for the operator to leave the robot cockpit to complete the procedure steps before removal of the coronary guide wire.

**Table 1:** Demographic and procedural characteristics

	Robotic PCI (n = 546)	Traditional PCI (n = 1654)	*P*
Age, y	58 (51-65)	60 (54-68)	<.001
Female	122 (22%)	347 (21%)	.51
Weight, kg	70 (64-79)	70 (65-79)	.68
Height, cm	165 (160-170)	165 (160-170)	.835
Acute coronary syndrome	145 (27%)	467 (28%)	.62
Hypertension	320 (59%)	1021 (62%)	.21
Diabetes	237 (43%)	751 (45%)	.43
Previous coronary bypass surgery	23 (5%)	38 (2%)	<.001
SYNTAX score	8 (6-13)	11 (8-18)	<.001
LVEF, %	60 (45-60)	50 (40-60)	<.001
Air kerma, mGy	698 (441-1194)	1268 (820-1934)	<.001
Dose-area product, Gy-cm^2^	3931 (2454-6912)	6624 (4340-10228)	<.001
Fluoroscopy time, min	4.5 (3.1-7.4)	6.5 (4.5-10.1)	<.001
Contrast volume, mL	110 (80-150)	150 (120-190)	<.001
Procedure time, min	27 (20-35)	35 (25-40)	<.001
Radial access	544 (99.6%)	1646 (99.5%)	.71
Intracoronary imaging	0	514 (31%)	.001
Atherectomy	0	62 (4%)	.01
Chronic total occlusion intervention	4 (0.7%)	134 (8%)	.008

Values are median (IQR) or n (%). Gy, Gray; LVEF, left ventricular ejection fraction; PCI, percutaneous coronary intervention; SYNTAX, Synergy between Percutaneous Coronary Intervention with Taxus and Cardiac Surgery.

### Statistical analysis

Procedure characteristics of R-PCI were summarized. The distributions of each of the primary endpoints were plotted against the case numbers in chronological sequence. Analyzing the slope and the best curve fits were identified using the best model. A change per subsequent procedure of <5 seconds for procedure time, <0.5 mL for contrast volume, and <5 seconds for FT were considered indicative of a flattening of the slope. The primary endpoint variables with R-PCI were compared to a matched cohort of T-PCI during the same follow-up period at the same institution. Matching was conducted using propensity scores and Greedy matching based on the following covariates: age, sex, history of hypertension, diabetes mellitus, previous myocardial infarction, previous percutaneous coronary intervention, previous coronary artery bypass graft surgery, dyslipidemia, smoking, left ventricular ejection fraction, body mass index, and SYNTAX score. The propensity model results are shown in Table 2.

**Table 2:** Propensity score model for robotic versus traditional PCI

	Estimate	Odds Ratio	95% Confidence Interval	*P*
Age	−0.02	0.98	0.97-0.99	<.001
Female	−0.08	0.86	0.67-1.1	.23
Hypertension	−0.11	0.9	0.72-1.12	.34
Diabetes	0.00	1	0.81-1.24	.99
Myocardial infarction	0.33	1.39	1.1-1.77	.007
PCI	0.18	1.2	0.91-1.59	.2
Hyperlipidemia	0.42	1.52	0.82-2.82	.18
Smoking	0.06	1.06	0.67-1.67	.8
CABG	1.19	1.19	1.87-5.83	<.001
LVEF	0.02	1.02	1.01-1.03	<.001
BMI	0.00	1	0.97-1.02	.8
SYNTAX score	−0.07	0.94	0.92-0.95	<.001
Intercept	0.09	–	–	.86

BMI, body mass index; CABG, coronary artery bypass graft surgery; LVEF, left ventricular ejection fraction; PCI, percutaneous coronary intervention; SYNTAX, Synergy between Percutaneous Coronary Intervention with Taxus and Cardiac Surgery.

In order to explore the trend over time, a simple moving average was displayed. The moving average of procedure times in the R-PCI and T-PCI cohorts were plotted. The standard learning curve model was fit defined by Y = aX^b^. The subsequent learning percentage was calculated as 100 × 2^b^. To explore a potentially more complex learning curve, locally estimated scatterplot smoothing (LOESS) or locally weighted regression models were used. Localized minimum and maximum were determined based on the slope of the curve.

## Results

A total of 546 consecutive R-PCI procedures and 1654 T-PCI procedures performed between December 2017 and July 2020 were analyzed. Table 1 shows the baseline and procedural characteristics of the study population. Although baseline characteristics of the R-PCI and T-PCI cohorts were comparable, T-PCI patients had more complex coronary anatomy as indicated by a higher SYNTAX score, were marginally but significantly older, and had lower left ventricular ejection fractions; hence, these findings may not apply to procedures deemed highly complex.

**Figure 1:**
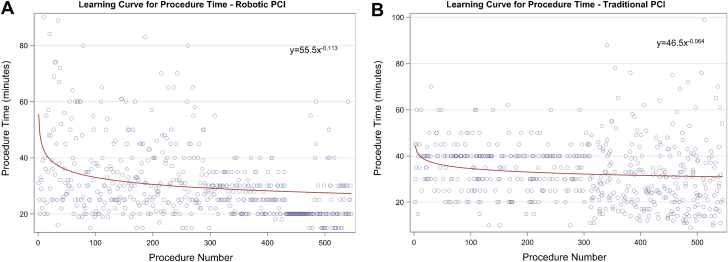
**Learning curve for procedure time.** The slope of the curve of procedure time plotted against procedure sequence for robotic PCI (A) and traditional PCI (B) demonstrated an exponential relationship with a significant change at procedure number 50. PCI, percutaneous coronary intervention.

For procedure time, the fitted model was y = 55.5x^-0.113^ as depicted in Figure 1. The learning curve percentage, derived from the reduction percentage, which denotes a decrease in unit metric with the doubling of number of cases, was 92.5%. The curve showed plateauing at approximately the 50th procedure (with future gains of <5 seconds per each additional procedure), with minor gains after the 112th procedure (<2 seconds per each additional procedure) (Figure 1A). Compared with the matched cohort of T-PCI (Figure 1B), based on the model, the increment in procedure time of R-PCI compared with T-PCI disappears at procedure 39.

**Figure 2:**
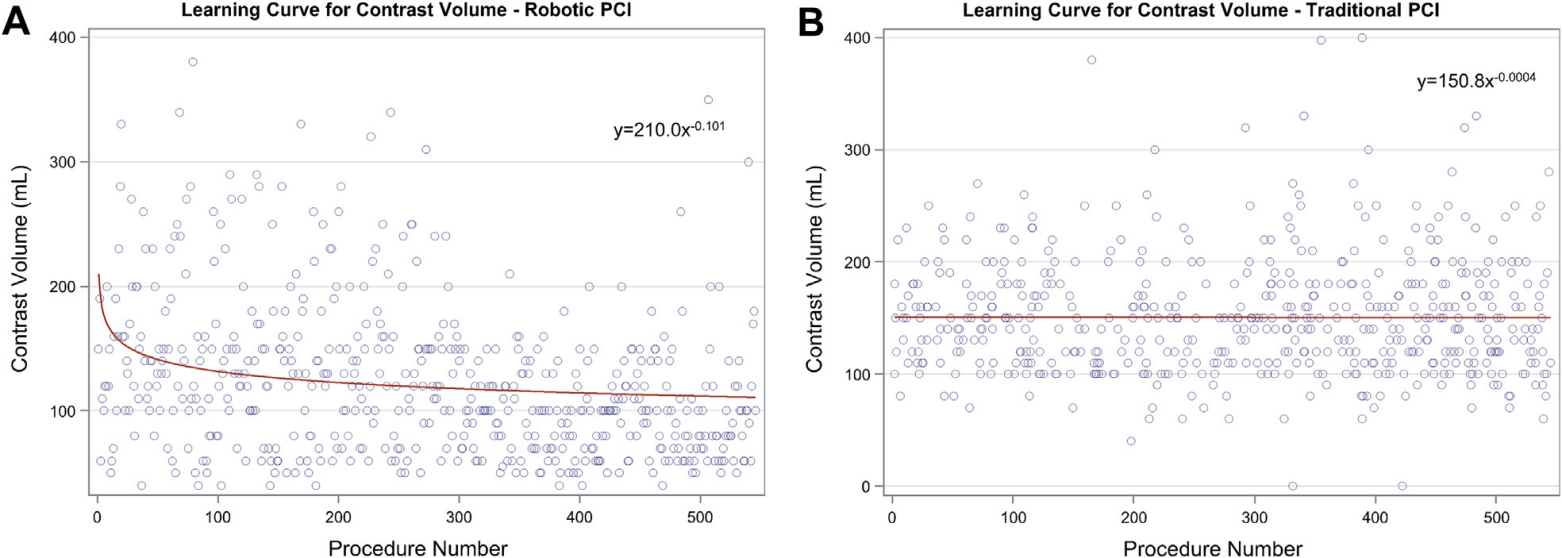
**Learning curve for contrast use.** The slope of the curve of contrast volume plotted against procedure sequence for robotic PCI (A) and traditional PCI (B) demonstrated an exponential relationship with a significant change at procedure number 30. PCI, percutaneous coronary intervention.

**Figure 3:**
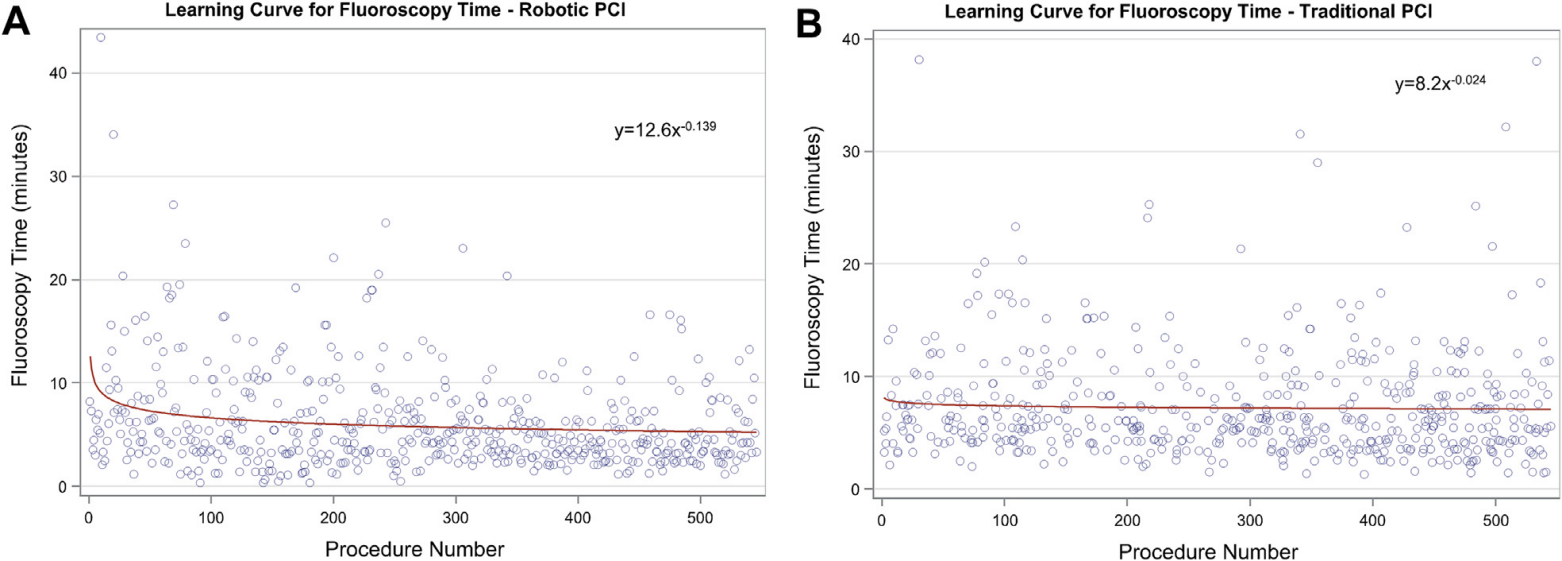
**Learning curve for fluoroscopy time.** The slope of the curve of fluoroscopy time plotted against procedure sequence for robotic PCI (A) and traditional PCI (B) demonstrated an exponential relationship with a significant change at procedure number 15. PCI, percutaneous coronary intervention.

Similar results were demonstrated for the volume of contrast used per procedure (Figure 2, learning percentage = 93.2%), with a <0.5 mL/procedure decrease after the 30th R-PCI procedure, marking the point where the slope flattened. FT (Figure 3, learning percentage = 93.2%) showed a <5 seconds/procedure decrement at procedure 15. In the matched cohort of T-PCI, the amount of contrast for R-PCI crossed that of T-PCI at procedure 27, and for FT, R-PCI crossed T-PCI at procedure 74. No learning curve was detected in the T-PCI cohort for either contrast volume or FT (learning percentage of >99.9% and 98.4%, respectively).

**Central Illustration:**
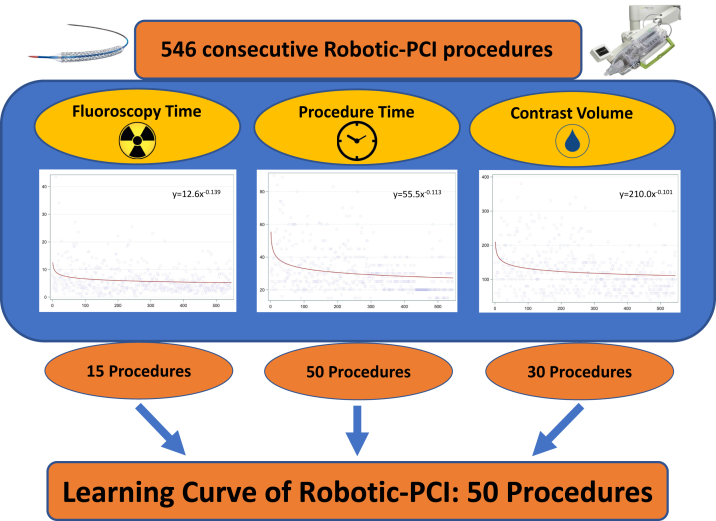
The "learning curve" as estimated by flattening of the slope of each metric is shown. By the 50th procedure, each of the 3 studied metrics demonstrate a flattening of the slope.

**Supplementary Figure S1:**
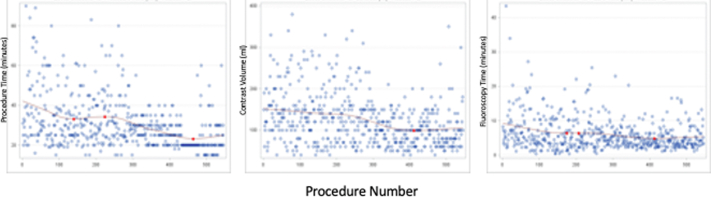


**Supplementary Figure S2:**
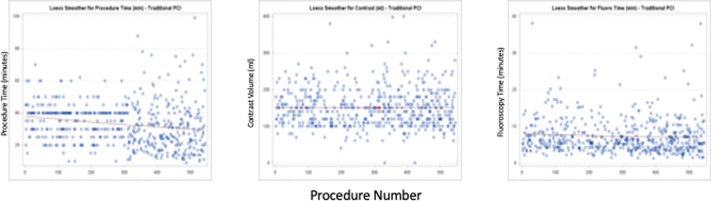


For procedure time, the LOESS model indicated change points detected at procedure numbers 139, 224, and 463, with the procedure time decreasing until procedure 139, leveling out until procedure 224, and then decreasing again until procedure 463, where it leveled out again (Supplemental Figure S1). For contrast volume, change points were detected at procedure number 412, where the amount of contrast leveled out. Similar to procedure time, 3 change points were detected at procedure numbers 174, 207, and 412. FT showed a decreasing trend until procedure 174, leveling out until procedure 207, and then decreasing slightly again until procedure 412, where it leveled out. In the matched cohort of T-PCI, there was no detected learning curve and no notable inflection points detected for procedure time, contrast volume, or FT, although there did appear to be a small linear improvement in procedure time over the time period (Supplemental Figure S2).

**Supplementary Figure S3:**
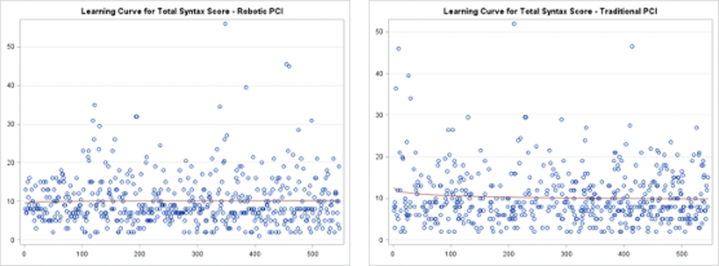


The traditional “learning curve model” was applied to the R-PCI group for SYNTAX score and for comparison in the matched T-PCI cohort. The SYNTAX score in the R-PCI arm was 10.0 ± 6.5 and 10.3 ± 6.5 in the matched T-PCI cohort, (*P* = .91) (Supplemental Figure S3).

Crossover from R-PCI to T-PCI occurred in 22 cases. All of these crossovers occurred in the first half of the experience, with last crossover at procedure 235. A majority (15 out of 22) of the crossovers occurred in the first quartile (Supplemental Figure S4). Three crossovers occurred because of the inability to cross the stenosis with a guide wire using the robotic console, 9 crossovers occurred because of an inability to advance the stent, and in 10 patients, manual manipulation of the guide catheter was needed. Procedural complications were infrequent, with 3 patients (0.6%) experiencing no-reflow in the R-PCI cohort and 8 patients (0.5%) with no-reflow in the T-PCI cohort, (*P* = .62). These were treated successfully with adjunctive pharmacotherapy.

## Discussion

Our data show that the learning curve for R-PCI appears to be approximately 50 procedures (Central Illustration). This approximation is based on the observations of the slope of the relationship between the outcome measure of procedure time, contrast volume, and FT when plotted against procedures in chronological sequence. Like many other procedures where a multidisciplinary approach is required, R-PCI involves learning a new method for the entire interventional team.^5^ The assistants and the technologists need to learn how to prepare the robotic arm at the time of initial patient preparation. They also need to learn how to load disposable hardware such as guide wires, balloons, and stent catheters onto the robotic arm with subsequent exchanges as necessary. This process of learning is certainly expected to increase the procedure duration, as observed in our data set and published earlier.^3^

For the other 2 outcome measures studied, contrast volume and FT, most of the learning occurs on the part of the primary operator. In the initial phases of the R-PCI experience, more contrast agent use is observed, although once again as the operator develops confidence with the robotic control system and the new ergonomics, contrast volume appears to plateau. The slope of FT follows a similar contour. The improvement in these metrics continues after these procedure points, indicating a continued learning process as experience grows.

The absence of crossover from R-PCI to nonrobotic percutaneous coronary intervention after the first half of the procedures likely also indicates an improvement in the confidence of the operator in the R-PCI system with development of techniques to troubleshoot procedural hurdles without the need to abandon the robotic setup. The crossover rates in our study were lower than those reported previously, likely because of case selection and differences in case complexity.^6^ The other metric that may affect these parameters, the complexity of the anatomic substrate as measured by SYNTAX score, did not show a significant change throughout the procedure sequence. This lack of significant change in the SYNTAX score over the study period of patients undergoing R-PCI makes the effect of a significant selection bias on the improvement in the primary outcome measures less likely. Although SYNTAX score among patients treated with R-PCI was similar to T-PCI, indicating absence of a large selection bias when the metrics were compared between R-PCI and T-PCI, some element of selection bias based on differences in unmeasured confounders is expected. The SYNTAX score in the R-PCI cohort indicates a noncomplex coronary lesion cohort, largely due to limitations of the robotic console; with expanding capabilities of the equipment, the application of R-PCI to complex coronary subsets is expected.

Radiation metrics such as FT, dose-area product, and air kerma as well as contrast utilization and procedure time were significantly lower in the R-PCI cohort compared to T-PCI. This likely is driven by the lower complexity of patients treated with R-PCI, although an independent beneficial effect of R-PCI on radiation metrics has been described earlier by Patel et al^3^ and may be responsible for this difference.

Our data provide the first learning curve description for R-PCI. The learning process appears to be reasonable in duration, especially for the multidisciplinary composite of the primary operator and team, as evidenced by procedure time, as well as the primary operator centric metrics of contrast volume used and FT. These data may be of value for institutional as well as individual operator policy development for processes such as credentialing.

Our findings are limited by the single-center nature of our cohort. Because all procedures were performed by 1 experienced operator, it would be improper to generalize these findings to multiple operators or to a less experienced cohort of operators and team members. Multiple operators performed T-PCI, and hence, the T-PCI metrics likely represent some heterogeneity, although this is not expected to affect the R-PCI learning curve results. The learning curve of R-PCI with the second operator with >10 years’ experience may be different from programs with technologist as the second operator. A registry-based analysis of learning curve domains is necessary to further identify the details of the learning process with R-PCI.

## Conclusion

The learning curve of R-PCI appears to be approximately 50 procedures, with a continuing improvement in the study metrics over the longer term.

## Figures and Tables

**Supplementary Figure S4:**